# A Mixture of Endocrine Disruptors and the Pesticide Roundup^®^ Induce Oxidative Stress in Rabbit Liver When Administered under the Long-Term Low-Dose Regimen: Reinforcing the Notion of Real-Life Risk Simulation

**DOI:** 10.3390/toxics10040190

**Published:** 2022-04-14

**Authors:** Periklis Vardakas, Aristidis S. Veskoukis, Danai Rossiou, Christos Gournikis, Theodora Kapetanopoulou, Vasiliki Karzi, Anca Oana Docea, Aristidis Tsatsakis, Demetrios Kouretas

**Affiliations:** 1 Department of Biochemistry and Biotechnology, University of Thessaly, 41500 Larissa, Greece; periklis_vardakas94@hotmail.com (P.V.); veskoukis@uth.gr (A.S.V.); danai.rossiou@gmail.com (D.R.); cgournikis@uth.gr (C.G.); theodora_kap@hotmail.com (T.K.); 2 Department of Nutrition and Dietetics, University of Thessaly, 42132 Trikala, Greece; 3 Department of Toxicology, Medical School, University of Crete, 71003 Heraklion, Greece; chemstud.vas2010@gmail.com (V.K.); tsatsaka@uoc.gr (A.T.); 4 Department of Toxicology, University of Medicine and Pharmacy of Craiova, 200349 Craiova, Romania; ancadocea@gmail.com

**Keywords:** redox biomarkers, redox status, oxidative stress, endocrine disruptors, pesticides, real-life exposure scenario, real-life risk simulation

## Abstract

Humans are exposed to xenobiotic mixtures daily through the long-term, low-dose regimen. Investigations designed to simulate this exposure profile approach the real-life risk simulation (RLRS) idea of modern toxicology. The purpose of the present study was to investigate the effects of 12-month exposure of New Zealand rabbits to a xenobiotic mixture comprising seven endocrine disruptors (EDs), which are chemical substances raising great concerns for human health, as well as the herbicide glyphosate, and its commercial formulation Roundup^®^, on blood and tissues redox status. It is reported herein that at the systemic level, the administration of the EDs mixture induced perturbations of blood redox homeostasis at 3 months, whereas at 6 and 12 months, it activated redox adaptations. Contrariwise, exposure to glyphosate and Roundup^®^, individually, caused mainly disturbances of blood redox equilibrium. At the tissue level, particularly in the liver, the administration of both the EDs mixture and Roundup^®^ induced oxidative stress, whereas glyphosate did not affect it. The RLRS notion appears to be confirmed through these findings. Indeed, the administration of the EDs mixture and Roundup^®^, under the long-term, low-dose regimen, elicited detrimental effects on the redox status of the liver, a crucial tissue with a valuable biological role in the detoxification of organisms from xenobiotics.

## 1. Introduction

Humans are exposed daily to many chemicals from multiple sources (e.g., workplace, diet, lifestyle products) and through different body routes (e.g., inhalation, skin contact, ingestion). Pesticides, preservatives, and synthetic compounds used in packaging and storage are admittedly the most encountered among these chemical substances. Pesticides comprise a class of compounds, divided into subclasses based on the pests they target and how they act (i.e., through repelling, destroying, or controlling pests) [1]. Their unprecedented use has marked the last century since the elevated consumer needs have demanded an increase in crop yields and food production [2]. Concurrently, human exposure rates have been elevated through the occupational environment, contaminated foods and water consumption, and direct contact with polluted air [3]. Bioaccumulation of pesticides and/or their metabolites in the mammalian tissues constitutes a chronic health risk factor [4]. Exposure to pesticides has been linked to respiratory diseases [5], cardiotoxicity [6], neurotoxicity [1], and carcinogenicity [7]. At the same time, several of them are suspected or even identified to act as endocrine disruptors (EDs), thereby modifying the normal function of the endocrine system [8].

Preservatives are natural or synthetic compounds that inhibit the growth of microorganisms and, therefore, are applied for the shelf-life extension of products in the food and pharmaceutical industries [9]. In the absence of a preservation condition, the aforementioned products are rapidly contaminated with microorganisms, resulting in spoilage and an increased risk of infection [10,11]. To be safely used, chemical preservatives must meet a series of requirements to demonstrate sufficient solubility, be efficient antimicrobial agents over food pH range, be cost-effective and easily applied to the foods, and, of course, not induce any toxicity [12]. Nevertheless, they can putatively cause adverse effects on human health. For instance, this is the case for parabens, which, although they are among the most popular preservatives worldwide, exert weak estrogenic activities [13,14]. In recent years, there has been a growing demand for preservative-free products. Consumers have developed negative perceptions of the potential adverse effects of synthetic preservatives associated with actions on the endocrine system [15].

Synthetic compounds that are used in food packaging, transfer, and storage, are also major factors for inducing toxicity in humans, owing to their tendency to accumulate in consumed products [16,17]. Phthalates and bisphenol A (BPA) represent some of the products that are produced at a higher rate globally [18], but, at the same time, their use has raised several concerns. The temporary interaction of phthalates with the polymer resin macromolecules is responsible for their migration from plastic cans to the surrounding environment, under the influence of heat and pH [19]. Correspondingly, the ester bond that links the molecules of BPA within the matrix is rapidly hydrolyzed under the influence of heat or direct contact with acidic or basic compounds. As a result, BPA contaminates foods and beverages [20]. The greatest concern about phthalates and BPA lies in their interference with the endocrine system, leading to toxic effects related to development and reproduction [19,21].

Based on the above, the potential health risks arising from daily exposure to low doses of EDs mixtures represent an area of great concern due to the modern lifestyle [22]. According to World Health Organization (WHO), an ED is defined as “an exogenous substance or mixture that alters function(s) of the endocrine system and consequently causes adverse health effects in an intact organism, or its progeny, or (sub)populations” [23]. Regarding EDs, their mechanism of action involves the mimicry or antagonism of natural hormones and the blockage of hormone-receptor interactions [24,25]. The adverse effects of the latter compounds include obesity and relevant diseases [26], perturbations of the reproductive system [27], immunotoxicity [28], and carcinogenicity [29,30]. Additionally, exposure to EDs during developmental periods can negatively affect the directly exposed individuals and future generations via the inherited epigenetic modifications, hence perpetuating the current problem and increasing its criticality [31].

The present study is part of a multidisciplinary approach to the novel notion that recently emerged in toxicology, inducing a perception shift in the ideas of this scientific area [32,33,34]. Most toxicological studies have investigated the toxic properties of xenobiotics administered acutely and individually at high, unrealistic doses. At the same time, chronic toxicity evaluations have also been conducted for individual chemicals. Nevertheless, humans are exposed daily to mixtures of such compounds at doses around or far below the current regulatory limits. As a result, assessing the biological or clinical outcomes of chemical mixtures administered under the long-term, low-dose regimen is rather the realistic approach, which has been insightfully mentioned as the real-life risk simulation (RLRS) or the real-life exposure scenario [35]. This concept appears to be common sense. For instance, the interactions between chemicals, occurring from their concurrent presence, may lead to potentiating, synergistic or antagonistic effects related to known or even unpredicted hazards [36]. Exposure time is also a crucial parameter for the toxicity onset along with the dose. Hence, the long-term, low-dose exposure regimen to mixtures of xenobiotics has been proposed as a new methodological approach [4,32]. Oxidative stress is a crucial and often primary outcome among the various surrogate endpoints, and its assessment can provide valuable insights [17,37,38].

The choice of the appropriate xenobiotics comprising a potentially examined mixture for its in vivo redox-altering properties is the challenge of investigating the real-life exposure scenario [4,17]. In this regard, the present study aimed to evaluate the effects of a xenobiotic mixture comprising endocrine-disrupting chemicals, as well as the effects of the herbicide Roundup^®^ and its active ingredient glyphosate, administered in a long-term, low-dose manner (i.e., in doses far below no adverse effect level, NOAEL), on rabbit blood and tissue redox status, through the measurement of an established battery of redox biomarkers [39,40]. A parameter that should be considered is that although the exposure of the animals to the xenobiotics examined in this study was chronic, it lasted only 12 months, a duration much lower than their proximate life expectancy, which is about 10 years [41]. The administered mixture was composed of the herbicide glyphosate, the chemicals methylparaben (MePB), butylparaben (BuPB), propylparaben (PrPB), and triclosan (TCS), which are preservatives or antimicrobial agents, and the synthetic compounds BPA and di-(2-ethylhexyl) phthalate (DEHP). In brief, glyphosate, a herbicide that targets the shikimic acid pathway present in plants and some microorganisms [42], is used worldwide to control weeds and vegetation in agricultural and non-agricultural areas, respectively and as a crop dryer to enhance farming yield [43]. Roundup^®^ is a commercial formulation of glyphosate mixed with water, colorants, and some surfactants, predominantly polyethoxylated tallow amine (POEA), added to enhance the uptake and translocation of the herbicide active ingredient [44]. Methyl-, butyl-, and propylparaben are esters of p-hydroxybenzoic acid, widely applied as antimicrobial preservatives in foods, cosmetics, medicines, and pharmaceuticals either alone or, in many cases, in combination with other parabens or antimicrobial agents [45,46]. Triclosan, a broad-spectrum antimicrobial agent, is utilized as an antiseptic and disinfectant in medical settings and as a preservative in personal care products [47]. BPA is a synthetic organic compound mainly used for the production of synthetic polymers but also as a stabilizer and antioxidant in the production of vinyl chloride and thermal paper [48,49]. Finally, DEHP, a well-known toxic substance for growth and reproduction, is used primarily as a plasticizer to produce polyvinyl chloride [50,51].

## 2. Materials and Methods

### 2.1. Animals

In this study, twenty clinically healthy, 2–3 months old, New Zealand rabbits were used. The New Zealand breed is often preferred for research activities because of the reduced aggression and fewer health problems compared to other breeds [52]. In detail, 10 male rabbits with an average weight of 2.94 kg (ranging from 2.5 to 3.25 kg) and 10 female rabbits with an average weight of 3.02 kg (ranging from 2.75 to 3.3 kg) were used. The rabbits were bred under a constant 12-h light/dark cycle and controlled temperature (23–24 °C).

### 2.2. Administration Schemes and Dosages of Xenobiotics

The EDs mixture was administered at doses equal to 1 × ADI and 10 × ADI, whereas glyphosate and Roundup^®^ were administered at a dose equal to 10 × ADI; ADI is defined as the acceptable daily intake expressed in mg/kg body weight/day. In consonance with the European Food Safety Authority (EFSA), the ADI for BPA has been assessed at 0.004 mg/kg body weight/day, for BuPB at 0.5 mg/kg body weight/day, for MePB and PrPB at 5 mg/kg body weight/day, for phthalates at 0.05 mg/kg body weight/day, and for glyphosate and Roundup at 0.5 mg/kg body weight/day. Finally, the ADI for TCS has been set by the Government of Canada at 0.08 mg/kg body weight/day.

### 2.3. Study Design

**Figure 1:**
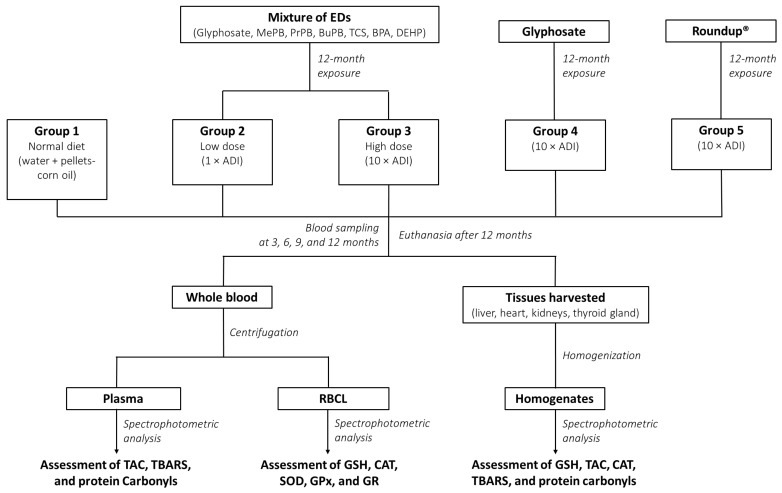
The study design. EDs: Endocrine disruptors; MePB: Methylparaben; PrPB: Propylparaben; BuPB: Butylparaben; TCS: Triclosan; BPA: Bisphenol A; DEHP: di-(2-ethylhexyl) phthalate; ADI: Acceptable daily intake; RBCL: Red blood cell lysate; GSH: Reduced form of glutathione; TAC: Total antioxidant capacity; CAT: Catalase; SOD: Superoxide dismutase; GPx: Glutathione peroxidase; GR: Glutathione reductase; TBARS: Thiobarbituric acid reactive substances.

The experimental animals were randomly assigned to 5 groups, consisting of 2 male and 2 female rabbits. The study design is illustrated in Figure 1. Group 1 (i.e., the control group) received a normal diet comprising water and pellets-corn oil with 5% ethanol/water. Groups 2 and 3 received the EDs mixture in two dose levels mixed with their food. In particular, the doses were equal to 1 × ADI and 10 × ADI, referred to hereafter as the low and high dose, LD and HD, respectively. Group 4 received glyphosate in a dose equal to 10 × ADI, and Group 5 received Roundup^®^ in a dose equal to 10 × ADI. All chemicals were dissolved in a 5% ethanol/water solution and administered once daily for 12 months. Upon the end of the experiment, the animals were humanely euthanized, under Xylapan/Narketan (2:1 *v*/*v*) anesthesia, by an injectable solution (i.e., Dolethal, 1 mL/kg body weight).

### 2.4. Blood and Tissues Collection and Handling

Blood was collected from cardiac puncture and immediately centrifuged (138× *g*, 10 min, 4 °C) to separate plasma from erythrocytes. Plasma was collected and stored in aliquots at −80 °C, until further analysis, while red blood cells were hemolyzed with distilled water (1:1 *v*/*v*). Subsequently, the samples were centrifuged (400× *g*, 15 min, 4 °C) and the supernatant (i.e., red blood cell lysate, RBCL) was collected and stored in aliquots at −80 °C. Liver, heart, left kidney, right kidney, and thyroid gland were excised, snapped-frozen in liquid nitrogen, and kept at −80 °C, until further analysis. For homogenization, the tissues were weighed and mixed with phosphate-buffered saline (PBS) containing a Roche cOmplete^™^ protease inhibitor cocktail tablet (Roche Diagnostics, Mannheim, Germany). The samples were transferred to Minilys personal homogenizer (Bertin Instruments, Montigny-le-Bretonneux, France), homogenized for 30 s at 5000 rpm, placed on ice for 20 s, and centrifuged (15,00× *g*, 5 min, 5 °C). The resulting supernatant (i.e., the tissue homogenate) was collected, divided into aliquots, and stored at −80 °C.

### 2.5. Protocols for the Determination of Blood and Tissue Redox Biomarkers

#### 2.5.1. Determination of GSH Concentration in RBCL and Tissues

GSH concentration was determined according to the method of Reddy et al. [53], as previously described [39]. At first, 400 μL of RBCL or 50 μL of tissue homogenate was mixed with 400 μL or 50 μL of 5% trichloroacetic acid (TCA), respectively, and centrifuged (15,00× *g*, 5 min, 5 °C). In the case of tissues, the resulting supernatant was collected and used as the biological sample for the assay. Contrariwise, in the case of RBCL, 300 μL of the supernatant was mixed with 90 μL of 5% TCA and centrifuged (15,00× *g*, 5 min, 5 °C). The resulting supernatant was collected and used as the biological sample for the assay. Regarding the assay, 20 μL of the biological sample, diluted 1:2 in PBS for tissue homogenates, was mixed with 660 μL of phosphate buffer (67 mM, pH = 7.95) and 330 μL of 5,5-dithiobis(2-nitrobenzoic acid) (DTNB) (1 mM). The samples were vortexed and incubated for 45 min in the dark at room temperature (RT), and the optical density was measured at 412 nm. GSH concentration was calculated based on the millimolar extinction coefficient of 2-nitro-5-thiobenzoate (TNB) (13.6 L/mmol/cm).

#### 2.5.2. Determination of CAT Activity in RBCL and Tissues

CAT activity was assessed based on a slightly modified method of Aebi [54]. In particular, 4 μL of RBCL, diluted 1:10 in PBS, or 40 μL of diluted tissue homogenate in PBS (i.e., 1:5 for heart and thyroid gland, 1:20 for liver, and 1:40 for kidneys) was added to 2991 μL or 2955 μL of phosphate buffer (67 mM, pH = 7.4), respectively. The samples were vortexed, incubated for 10 min at 37 °C, and transferred in a UV quartz cuvette, wherein 5 μL of 30% hydrogen peroxide (H_2_O_2_) was added. The decrease in optical density was monitored at 240 nm for 2 min. CAT activity was estimated based on the molar extinction coefficient of H_2_O_2_ (40 L/mol/cm).

#### 2.5.3. Determination of SOD Activity in RBCL

SOD activity was determined according to the method of Oberley and Spitz [55]. More specifically, 100 μL of RBCL, diluted 1:100 in PBS, was mixed with 800 μL of a master mix comprising diethylenetriaminepentaacetic acid (DETAPAC) (1.34 mM) in potassium phosphate buffer (0.05 M, pH = 7.8), nitro blue tetrazolium (NBT) (2.24 mM) in potassium phosphate buffer, and xanthine (1.18 mM) in potassium phosphate buffer. The samples were vortexed, and 100 μL of xanthine oxidase (60 mU) in DETAPAC was added. The change in optical density was monitored at 560 nm for 1.5 min. The percentage inhibition of NBT calculated SOD activity.

#### 2.5.4. Determination of GPx Activity in RBCL

GPx activity was evaluated according to the protocol of Flohé and Günzler [56], as formerly reported [39]. In particular, 100 μL of RBCL, diluted 1:100 in PBS, was mixed with 500 μL of phosphate buffer (100 mM, pH = 7), 100 μL of GR (0.24 U), and 100 μL of GSH (10 mM) and incubated for 10 min at RT. Then, the samples were transferred in a cuvette, 100 μL of NADPH (1.5 mM) in 0.1% NaHCO_3_ was added, and the NADPH consumption was monitored at 340 nm for 3 min. Finally, 100 μL of tert-butyl hydroperoxide (12 mM) was added, and the change in optical density was monitored at 340 nm for 3 min. GPx activity was calculated using the molar extinction coefficient of NADPH (6.22 L/mmol/cm).

#### 2.5.5. Determination of GR Activity in RBCL

GR activity was assessed according to the protocol of Smith et al. [57], as previously reported [39]. More precisely, 700 μL of phosphate buffer (200 mM, 1 mM EDTA, pH = 7.5), 250 μL of DTNB (3 mM), and 50 μL of β-NADPH (2 mM) were mixed, vortexed, and transferred in a cuvette. Then, 50 μL of GSSG (20 mM) in phosphate buffer and 25 μL of RBCL, diluted 1:100 in PBS, were sequentially added, and the change in optical density was monitored at 412 nm for 1 min.

#### 2.5.6. Determination of TAC Levels in Plasma and Tissues

TAC levels were evaluated based on the protocol of Janaszewska and Bartosz [58]. More elaborately, 20 μL of plasma or 40 μL of diluted tissue homogenate in PBS (i.e., 1:5 for the thyroid gland and 1:10 for liver, heart, and kidneys) was mixed with 480 μL or 460 μL of phosphate buffer (10 mM, pH = 7.4), respectively, and, immediately, 500 μL of 2,2-diphenyl-1-picrylhydrazyl radical (DPPH^•^) solution (0.1 mM) was added. The samples were vortexed, incubated for 1 h in the dark at RT, and centrifuged (15,00× *g*, 3 min, 25 °C). Finally, the optical density was measured at 520 nm. TAC levels were expressed as the mmol of DPPH^•^ reduced to the corresponding hydrazine by the antioxidant compounds present in plasma or tissue homogenates.

#### 2.5.7. Determination of TBARS Levels in Plasma and Tissues

TBARS levels were determined by a slightly modified method by Keles et al. [59]. More specifically, 100 μL of plasma or 50 μL of diluted tissue homogenate in PBS (i.e., 1:2 for heart and thyroid gland and 1:5 for liver and kidneys) was mixed with 500 μL of Tris-HCl (200 mM, pH = 7.4) and 500 μL of 35% TCA and incubated for 10 min at RT. After that, 1 mL of sodium sulfate (Na_2_SO_4_) (2 M) and thiobarbituric acid (TBA) (55 mM) solution was added, and the samples were placed in a water bath for 45 min at 95 °C. After incubation, the samples were cooled on ice for 5 min, 1 mL of 70% TCA was added, and the samples were centrifuged (11,20× *g*, 3 min, 25 °C). The resulting supernatant was used to measure the optical density at 530 nm. TBARS levels were calculated by applying the molar extinction coefficient of malonyl dialdehyde (ΜDA) (156,000 L/mol/cm).

#### 2.5.8. Determination of Protein Carbonyls Concentration in Plasma and Tissues

Protein carbonyls concentration was evaluated by the method of Patsoukis et al. [60], as previously described [39]. In detail, 50 μL of plasma or diluted tissue homogenate in PBS (i.e., 1:2 for all tissues) was mixed with 50 μL of 20% TCA and vortexed. The samples were placed on ice for 15 min and then centrifuged (15,00× *g*, 5 min, 4 °C). The supernatant was discarded, and 500 μL of 2,4-dinitrophenylhydrazine (DNPH) (10 mM) in hydrochloric acid (HCl) (2.5 N) or 500 μL of HCl (2.5 N) was added to the samples or blanks, respectively. The samples were incubated for 1 h in the dark at RT, and during this process, they were vortexed intermittently every 15 min. Then, the samples were centrifuged (15,00× *g*, 5 min, 4 °C), the supernatant was discarded, and 1 mL of 10% TCA was added. The samples were vortexed and centrifuged (15,000× *g*, 5 min, 4 °C). The supernatant was discarded, and 1 mL of ethanol and ethyl acetate (1:1 *v*/*v*) solution was added. The samples were vortexed and centrifuged (15,00× *g*, 5 min, 4 °C). This step was repeated twice. Afterward, 1 mL of urea (5 M, pH = 2.3) was added, and the samples were vortexed and incubated at 37 °C for 15 min. Then, the samples were centrifuged (15,00× *g*, 3 min, 4 °C), and the optical density was measured at 375 nm. Protein carbonyls levels were quantified based on the millimolar extinction coefficient of DNPH (22 L/mmol/cm).

### 2.6. Estimation of Total Protein and Hemoglobin Concentrations

Τhe values of the redox biomarkers of plasma and tissue homogenates were normalized to total protein concentration, estimated by the Bradford assay [61]. Briefly, the determination of total protein concentration was based on a standard curve created by solutions with known concentrations of bovine serum albumin that served as a standard protein. On the contrary, the values of the redox biomarkers of RBCL were normalized to hemoglobin concentration, determined through hemiglobincyanide method using a commercially available kit (Dutch Diagnostics, Zutphen, Netherlands). Each assay was performed in triplicates.

### 2.7. Statistical Analysis

The data obtained from blood were analyzed through two-way ANOVA, followed by Tukey’s post hoc test for pairwise comparisons. The data obtained from tissues were analyzed using one-way ANOVA, followed by Tukey’s post hoc test to compare the means between different experimental groups. All results were expressed as mean ± standard error of the mean (SEM), and the differences were considered significant at *p* < 0.05. Statistical analyses were performed using SPSS software, version 20.0 (SPSS Inc., Chicago, IL, USA).

## 3. Results

### 3.1. Effects of Chemicals on Blood Redox Biomarkers after 3 Months of Exposure

**Figure 2:**
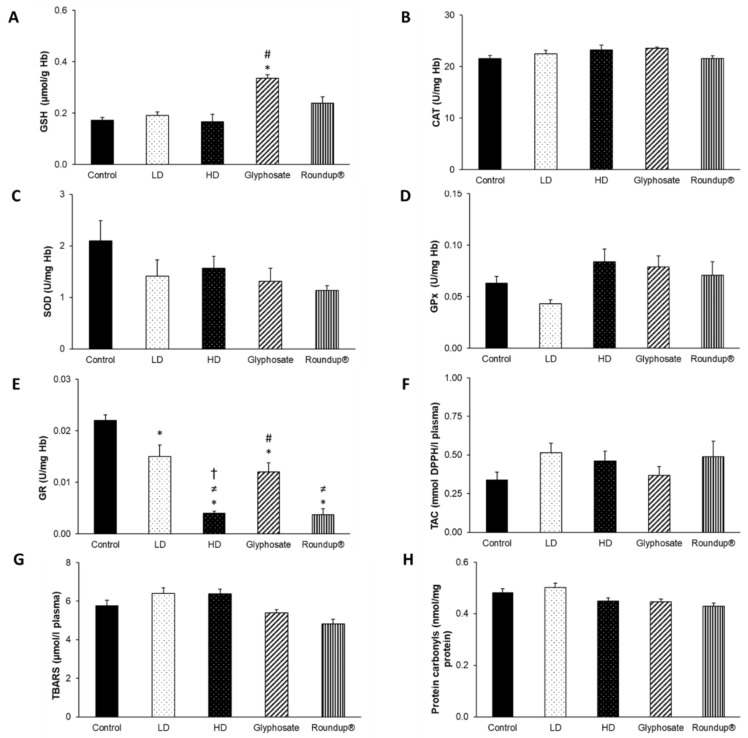
The effects of 3-month exposure of New Zealand rabbits to the EDs mixture, glyphosate, and Roundup^®^ on blood GSH concentration (**A**), CAT activity (**B**), SOD activity (**C**), GPx activity (**D**), GR activity (**E**), TAC levels (**F**), TBARS levels (**G**), and protein carbonyls concentration (**H**). *: Statistically significant difference compared to the control group. #: Statistically significant difference compared to the HD group. ≠: Statistically significant difference compared to LD group. †: Statistically significant difference compared to glyphosate group.

After 3 months of exposure to the EDs mixture, glyphosate, and its commercial formulation Roundup^®^, disturbances of blood redox equilibrium were observed. In particular, a statistically significant increase in GSH concentration was detected in the glyphosate group compared to the control and HD groups (Figure 2A). Furthermore, GR activity decreased after all administrations compared to the control group (Figure 2E). Notably, GR activity was also decreased in the HD group compared to LD and glyphosate groups, in the Roundup^®^ group compared to the LD group. In contrast, it was increased in the glyphosate group compared to the HD group. No other significant differences were detected (Figure 2B–D,F–H).

### 3.2. Effects of Chemicals on Blood Redox Biomarkers after 6 Months of Exposure

**Figure 3:**
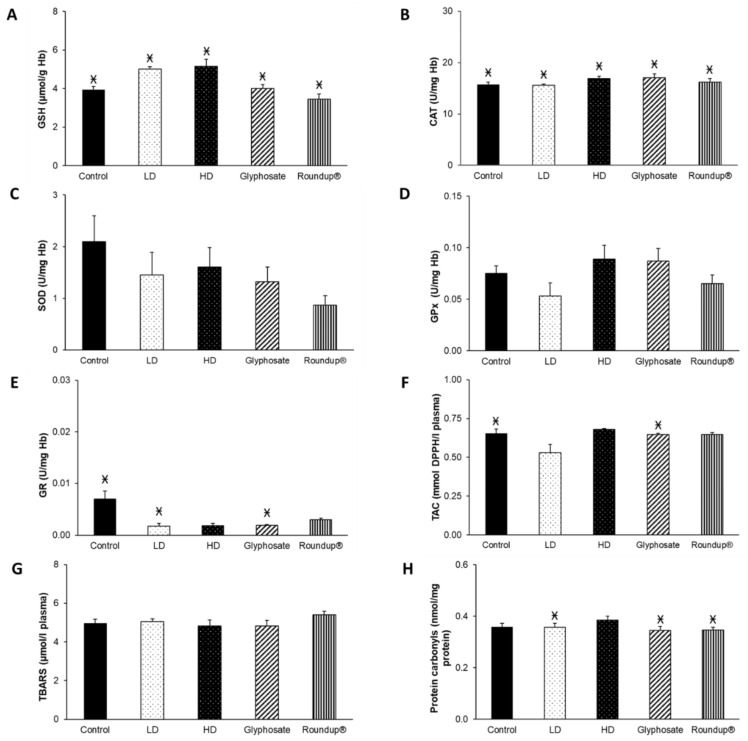
The effects of 6-month exposure of New Zealand rabbits to the EDs mixture, glyphosate, and Roundup^®^ on blood GSH concentration (**A**), CAT activity (**B**), SOD activity (**C**), GPx activity (**D**), GR activity (**E**), TAC levels (**F**), TBARS levels (**G**), and protein carbonyls concentration (**H**). Ӿ: Statistically significant difference within the same group compared to 3 months.

Following the 6-month exposure, no significant differences were observed between the different experimental groups. Nevertheless, there were significant differences between the different time points within the same group. GSH concentration showed a statistically significant increase at all experimental groups compared to the corresponding measurements at 3 months (Figure 3A). Additionally, CAT activity was statistically decreased in all groups compared to the 3 months (Figure 3B). Regarding GR activity, it was decreased after all treatments, except for HD and Roundup^®^ groups, as compared to 3 months (Figure 3E). Moreover, TAC levels were statistically increased in the control and glyphosate groups compared to 3 months (Figure 3F). Protein carbonyls concentration was statistically decreased in LD, glyphosate, and Roundup^®^ groups compared to 3 months (Figure 3H). SOD and GPx activities and TBARS levels remained unaffected (Figure 3C,D,G).

### 3.3. Effects of Chemicals on Blood Redox Biomarkers after 9 Months of Exposure

**Figure 4:**
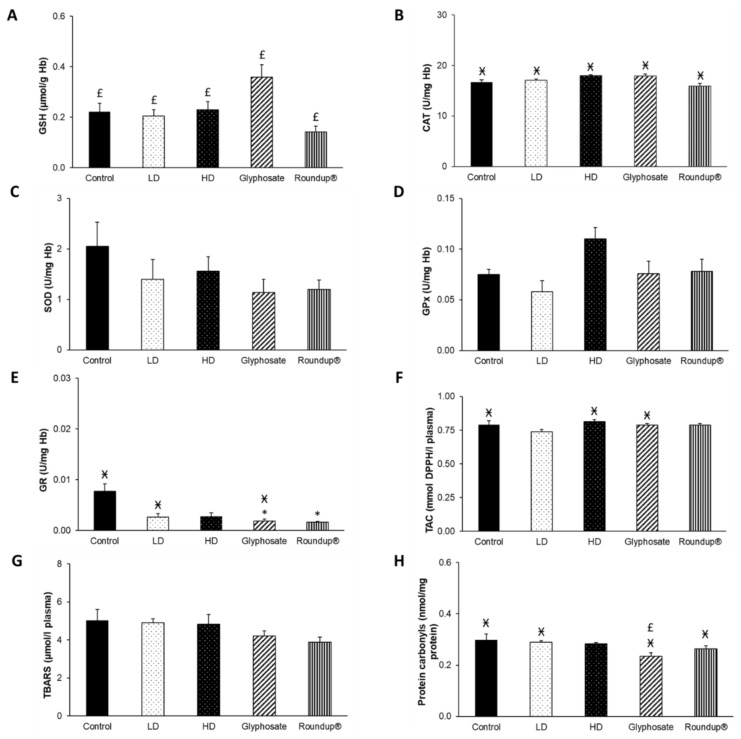
The effects of 9-month exposure of New Zealand rabbits to the EDs mixture, glyphosate, and Roundup^®^ on blood GSH concentration (**A**), CAT activity (**B**), SOD activity (**C**), GPx activity (**D**), GR activity (**E**), TAC levels (**F**), TBARS levels (**G**), and protein carbonyls concentration (**H**). *: Statistically significant difference compared to the control group. Ӿ: Statistically significant difference within the same group compared to 3 months. £: Statistically significant difference within the same group compared to 6 months.

Similar to the results obtained after 6 months of exposure, significant differences were observed mainly within the same group between different time points. Specifically, GSH concentration was statistically decreased in all groups in relation to the measurements obtained at 6 months (Figure 4A). Furthermore, a significant decrease in CAT activity was observed in all groups compared to 3 months (Figure 4B). Moreover, GR activity was statistically significantly decreased in the glyphosate and Roundup^®^ groups compared to the control group (Figure 4E). In contrast, it was decreased at the control, LD, and glyphosate groups compared to 3 months. A statistically significant increase was observed in TAC levels in the control, HD, and glyphosate groups, compared to 3 months (Figure 4F). The concentration of protein carbonyls was statistically decreased in all groups, except for the HD group, compared to 3 months (Figure 4H). It is noteworthy that in the glyphosate group, the concentration of protein carbonyls was also decreased compared to the 6 months. No significant differences were observed in SOD and GPx activities and in TBARS levels (Figure 4C,D,G).

### 3.4. Effects of Chemicals on Blood Redox Biomarkers after 12 Months of Exposure

**Figure 5:**
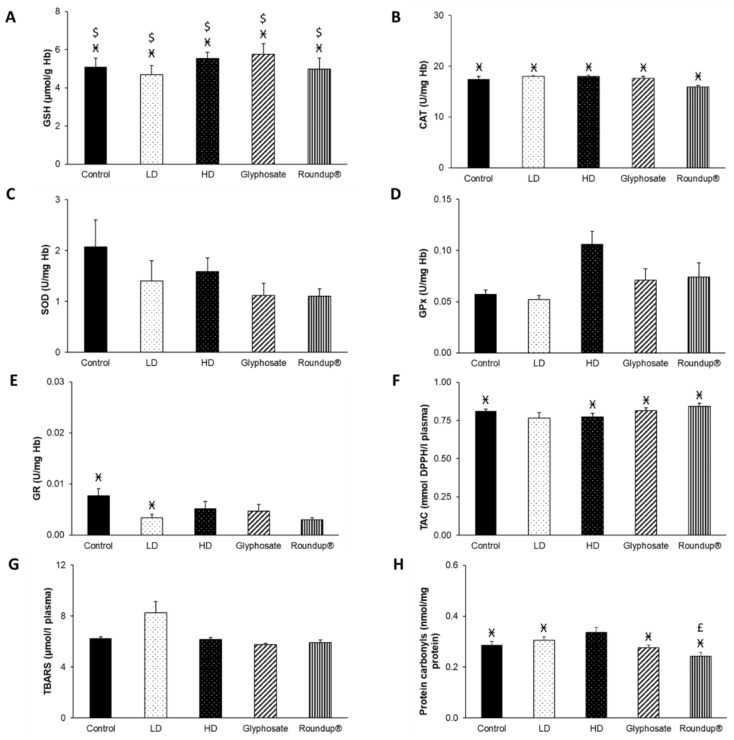
The effects of 12-month exposure of New Zealand rabbits to the EDs mixture, glyphosate, and Roundup^®^ on blood GSH concentration (**A**), CAT activity (**B**), SOD activity (**C**), GPx activity (**D**), GR activity (**E**), TAC levels (**F**), TBARS levels (**G**), and protein carbonyls concentration (**H**). Ӿ: Statistically significant difference within the same group compared to 3 months. £: Statistically significant difference within the same group compared to 6 months. $: Statistically significant difference within the same group compared to 9 months.

After 12 months of exposure, statistically significant differences were observed in the measured redox biomarkers only between the different time points. GSH concentration was statistically increased in all groups compared to the corresponding measurements at 3 and 9 months (Figure 5A). In terms of CAT activity, a statistically significant decrease was observed in all groups compared to the 3 months (Figure 5B). GR activity was decreased in the control and LD groups compared to 3 months (Figure 5E). A statistically significant increase was detected in TAC levels in all groups, compared to 3 months, except for the LD group (Figure 5F). The concentration of protein carbonyls was statistically decreased in all groups compared to the 3 months, except for the HD group. Moreover, it was also decreased in the Roundup^®^ group compared to 6 months (Figure 5H). SOD and GPx activities and TBARS levels were not altered (Figure 5C,D,G).

### 3.5. Effects of Chemicals on Tissues Redox Biomarkers after 12 Months of Exposure

**Figure 6:**
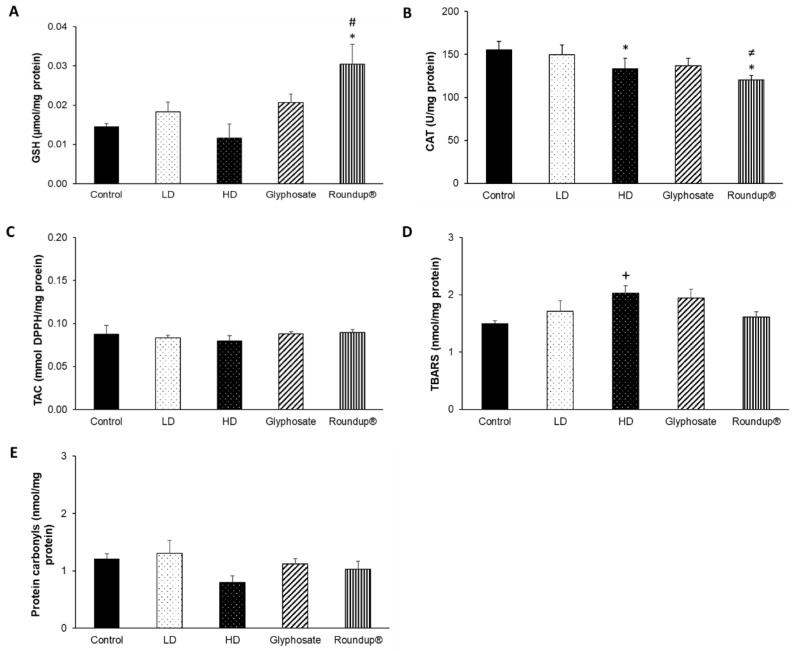
The effects of 12-month exposure of New Zealand rabbits to the EDs mixture, glyphosate, and Roundup^®^ on liver GSH concentration (**A**), CAT activity (**B**), TAC levels (**C**), TBARS levels (**D**), and protein carbonyls concentration (**E**). *: Statistically significant difference compared to the control group. #: Statistically significant difference compared to the HD group. ≠: Statistically significant difference compared to the LD group. +: Trend compared to the control group (*p* = 0.06).

Following the 12-month exposure to the EDs mixture, glyphosate, and its commercial formulation Roundup^®^, the only affected tissue was the liver. Regarding the effects of HD of the EDs mixture, CAT activity (Figure 6B) was statistically decreased compared to the control group, and, at the same time, a trend of increased TBARS levels (Figure 6D) was detected. On the contrary, LD did not affect the redox biomarkers tested. Of note, the 12-month administration of Roundup^®^ induced a statistically significant increase in GSH concentration (Figure 6A) compared to the control and HD groups, along with a statistically significant decrease in CAT activity compared to the control and LD groups (Figure 6B). Contrariwise, the 12-month exposure to glyphosate did not affect the levels of the measured redox biomarkers. TAC levels (Figure 6C) and protein carbonyls concentration (Figure 6E) remained unaffected.

**Figure 7:**
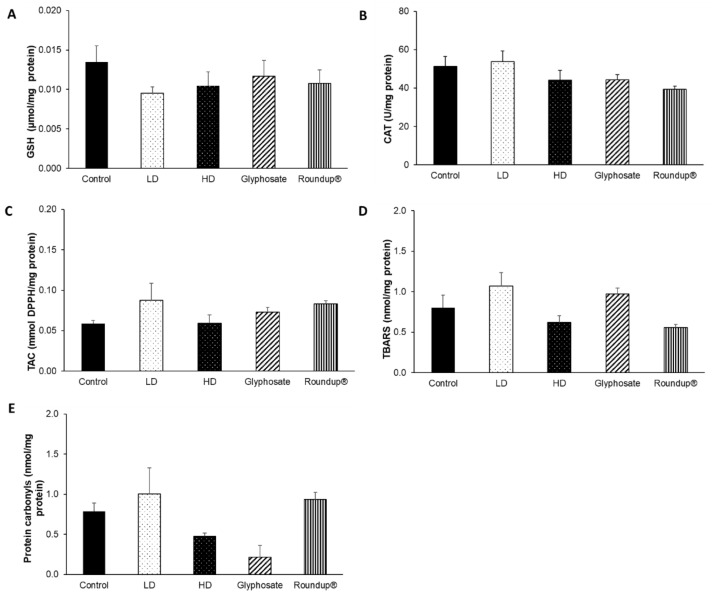
The effects of 12-month exposure of New Zealand rabbits to the EDs mixture, glyphosate, and Roundup^®^ on heart GSH concentration (**A**), CAT activity (**B**), TAC levels (**C**), TBARS levels (**D**), and protein carbonyls concentration (**E**).

**Figure 8:**
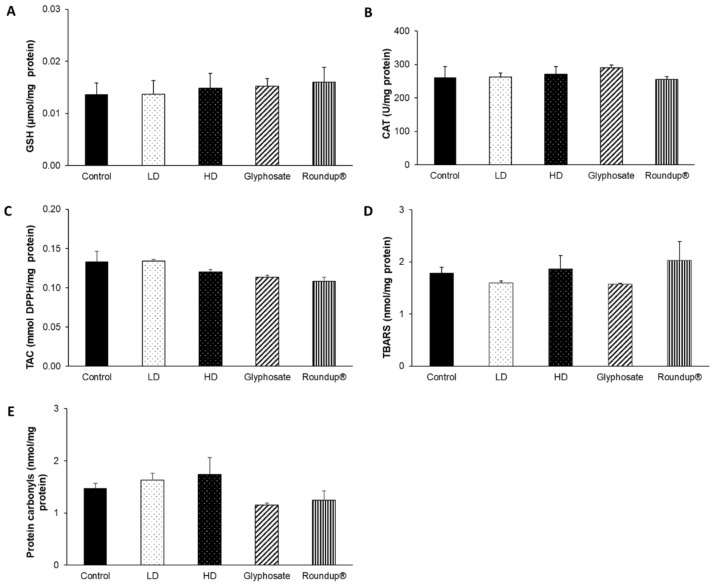
The effects of 12-month exposure of New Zealand rabbits to the EDs mixture, glyphosate, and Roundup^®^ on right kidney GSH concentration (**A**), CAT activity (**B**), TAC levels (**C**), TBARS levels (**D**), and protein carbonyls concentration (**E**).

**Figure 9:**
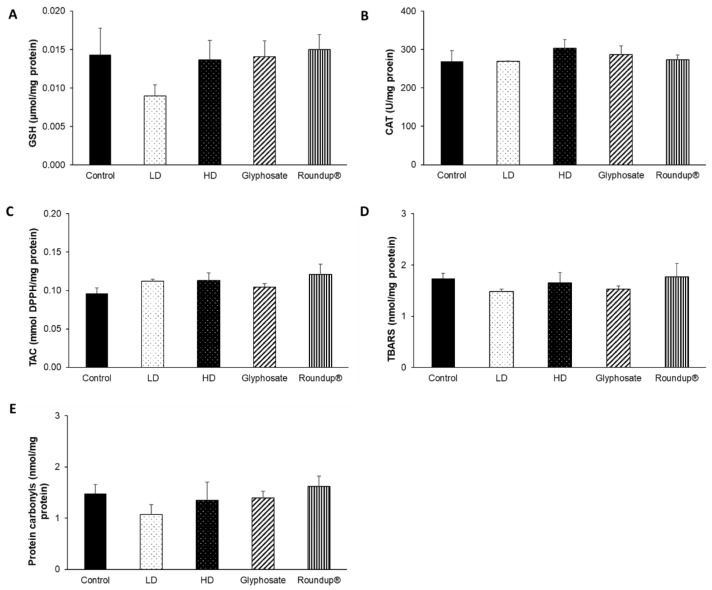
The effects of 12-month exposure of New Zealand rabbits to the EDs mixture, glyphosate, and Roundup^®^ on left kidney GSH concentration (**A**), CAT activity (**B**), TAC levels (**C**), TBARS levels (**D**), and protein carbonyls concentration (**E**).

**Figure 10:**
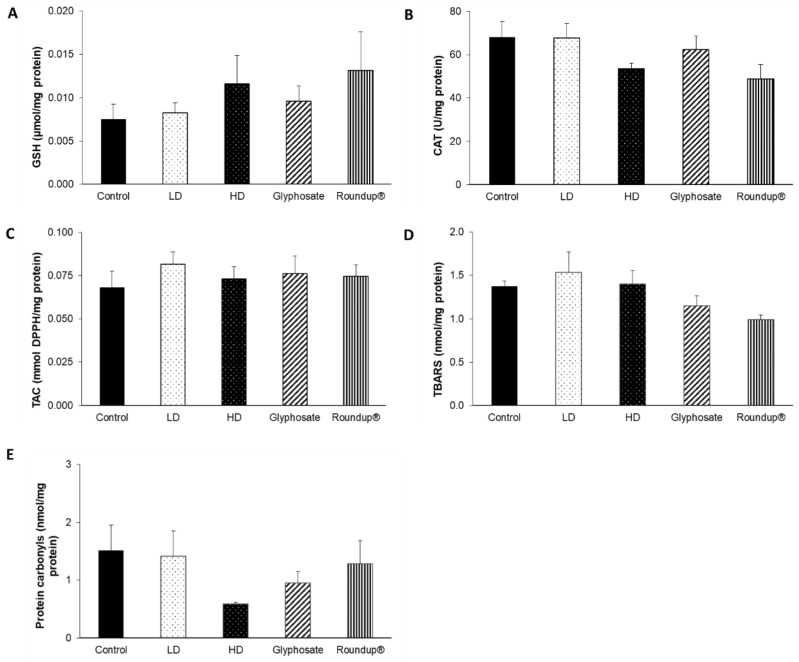
The effects of 12-month exposure of New Zealand rabbits to the EDs mixture, glyphosate, and Roundup^®^ on thyroid gland GSH concentration (**A**), CAT activity (**B**), TAC levels (**C**), TBARS levels (**D**), and protein carbonyls concentration (**E**).

None of the other examined tissues, namely the heart (Figure 7), right kidney (Figure 8), left kidney (Figure 9), and thyroid gland (Figure 10), was affected, in terms of their redox status, following the 12-month exposure to the examined xenobiotics.

## 4. Discussion

The main objective of the present study was to assess the effects of a chemical mixture composed of EDs, the herbicide Roundup^®^, and its active ingredient glyphosate, on the redox status of blood and tissues of New Zealand rabbits, after 12 months of daily exposure. The results obtained from the evaluation of redox biomarkers in blood demonstrated that the 12-month exposure to both the LD and HD of the EDs mixture activated useful antioxidant adaptations, an assertion supported by the increase in GSH levels and the reduction in protein carbonyl levels. On the contrary, the 12-month exposure to glyphosate and Roundup^®^ induced perturbations of blood redox homeostasis. However, in most cases, all administration schemes had the same motif of action at the same time point, with some exceptions, since no significant differences were detected compared to the control group. Contrariwise, the results obtained from the evaluation of tissues redox biomarkers dictate that the HD of the EDs mixture induced oxidative stress in the liver, as shown by the decrease in CAT activity and the trend of increasing TBARS levels. Similarly, the individually administered Roundup^®^ acted as an oxidizing agent since it decreased CAT activity and elevated GSH levels, thereby inducing detrimental effects on liver redox homeostasis. Of note, the inconsistencies observed between the effects on blood and tissue redox status indicate the complexity characterizing living organisms. The disturbances of liver redox equilibrium caused by chronic exposure to both the HD of EDs mixture and Roundup^®^ imply that the notion named RLRS appears to be confirmed in this particular experimental setting.

Regarding study parameters (i.e., the animal model and exposure time), rabbits and humans are biological systems characterized by high phylogenetic similarity. As a result, rabbits represent a reliable animal model for simulating human pathologies [41,62]. In terms of the exposure duration, 12 months is a rather short time interval compared to the life expectancy of New Zealand rabbits (i.e., 10 years), contrary to a previous relevant study, wherein a different xenobiotic mixture was administered to rats basically for their whole life (i.e., 18 months) [17]. Nevertheless, even under this limitation, the liver, a crucial tissue for xenobiotic detoxification, suffers from oxidative stress due to the chronic administration of both the HD of the mixture and Roundup^®^ under the long-term, low-dose regimen.

Recent studies suggest that xenobiotics are potentially more harmful when they are components of a chemical mixture than when administered individually [63]. Therefore, studying chemical mixtures is a realistic and physiologically relevant approach [32]. The design of the present study simulates the real-life exposure scenario. It attempts to provide new evidence for the need to formulate regulations and policies regarding the cumulative risk assessment [35,64]. Previous studies have assessed the effects of a different chemical mixture comprising routinely encountered xenobiotics, among which glyphosate, butylparaben, and BPA, on rat blood and tissues redox status, concluding that the specific mixture induced useful redox adaptations after 6 and 12 months of administration [17,37,38]. On the contrary, the prolonged administration for 18 months, a period corresponding to the whole life of the experimental animals, disrupted redox homeostasis and caused detrimental effects both on blood and tissues redox biomarkers [17].

In the present study, after 3 months of exposure to LD, HD, and Roundup^®^, blood redox status was adversely affected, indicated by the decrease in GR activity compared to the control group. In the case of glyphosate, although GR activity was decreased, GSH concentration was increased compared to the control and HD groups, presumably as an adaptive mechanism to protect macromolecules from oxidative damage. After 6 months of exposure, though, the blood antioxidant arsenal was activated to cope with possible impairments in redox homeostasis. GSH concentration was increased in all experimental groups, probably to compensate for the significant decrease in CAT activity [65,66]. Furthermore, TAC levels were increased in the control and glyphosate groups. At the same time, the increase in GSH concentration contributed to the decrease in protein carbonyls concentration at the LD, glyphosate, and Roundup^®^ groups, thus preventing oxidative stress. Interestingly, after 9 months of exposure, a significant decrease in GR activity was observed in the glyphosate and Roundup^®^ groups compared to the control group. Blood antioxidant mechanisms were adversely affected, except for the glyphosate group, wherein an adaptive mechanism was detected. With respect to GSH, its concentration was decreased in all groups compared to 6 months. The depletion of GSH pools could be attributed to either the elevated levels of reactive species, hence, converting GSH to its oxidized form (GSSG), or the decreased activity of GR, the enzyme responsible for GSH regeneration from GSSG. The alterations in the intracellular GSH/GSSG equilibrium are considered a key determinant of redox status. As a result, the depletion of GSH pools might contribute to the emergence of oxidative stress [67]. Nevertheless, in the case of glyphosate, GSH concentration was decreased as an adaptive mechanism to counteract the oxidative protein modifications, as depicted by the decreased protein carbonyls concentration in comparison with the 6 months. Concerning the 12-month exposure, it seems that the antioxidant mechanisms of blood have recovered, as shown by the increase in GSH concentration in all groups compared to 9 months. Exposure to LD and HD of the EDs mixture for 3 months disrupted blood redox equilibrium by decreasing GR activity. Still, they did not cause any other systemic effects at the subsequent time points. Additionally, exposure to glyphosate for 3 months activated antioxidant adaptations, as depicted by the increase in GSH concentration, but caused disturbances of redox equilibrium after 9 months, as shown by the reduction in GR activity. Finally, exposure to Roundup^®^ for 3 and 9 months induced perturbations of redox homeostasis, as depicted by the decrease in GR activity.

In its pure form or mixed with various adjuvants in commercial formulations, the harmful outcomes of glyphosate have been the main research topic in the recent past. Nonetheless, studies focusing on systemic effects are generally lacking [68]. The dosage and time of exposure are decisive factors for the manifestation of detrimental effects. At the same time, short-term exposure to low doses of glyphosate might activate the antioxidant arsenal of blood to prevent oxidative damage. As a case in point, exposure of Wistar rats to low levels of glyphosate that are considered safe for humans for 28 consecutive days activated blood antioxidant mechanisms (i.e., GPx activity), thus protecting them from lipid peroxidation [69]. In broad outline, commercial formulations of glyphosate, such as Roundup^®^, exerted stronger adverse effects than the individual active substance, a phenomenon attributed to the enhanced toxicity of surfactants contained [70]. For instance, a glyphosate-based herbicide induced oxidative stress in the blood of male Wistar rats following an 8-week administration due to GSH depletion, the decreased activity of important antioxidant enzymes, and the advanced lipid peroxidation [71]. Furthermore, in a comparative study, exposure of male albino rats to Roundup^®^ for 3 months induced oxidative stress, an argument supported by the diminished GSH reservoirs, the decrease in CAT activity, and the promotion of oxidative modifications of lipids. On the contrary, the administration of glyphosate individually caused disturbances of blood redox homeostasis only at high doses, as denoted by the decrease in GSH levels and the elevation in lipid peroxidation byproducts [72]. Although the accurate chemical composition of Roundup^®^ remains undisclosed for regulatory reasons, POEA is widely recognized as the major non-ionic surfactant added to fulfill the aforementioned purpose [73]. Numerous in vitro and in vivo studies have investigated the toxicity profile of POEA, incriminating the specific compound or its synergy with glyphosate for the pernicious effects of glyphosate-based formulations [74,75,76,77,78]. For that reason, in recent years, there has been a progressive transition to the use of less toxic surfactants within the European Union [70].

After the 12-month exposure, the only tissue that was affected was the liver, whose redox homeostasis was probably disrupted by the HD of the mixture and Roundup^®^. This finding is critical, considering that the liver is particularly susceptible to xenobiotic damage due to its central role in the metabolism and detoxification of xenobiotics [79]. In particular, the administration of the chemical mixture at HD caused a decrease in CAT activity. It is well established that CAT and SOD are the predominant antioxidant enzymes implicated in the elimination of reactive species formed during the bioactivation of xenobiotics in the liver [80]. Therefore, the decrease in CAT activity could lead to the accumulation of H_2_O_2_ that contributes to the generation of potent oxidants, such as hydroxyl radical (OH^•^), through the Fenton reaction [81]. In this way, lipid peroxidation was promoted, as shown by the increasing tendency of TBARS levels, inducing oxidative stress in the liver.

Although the chemical substances of the present study have not been previously examined in a mixture form, toxicological investigations have demonstrated their ability to act individually as oxidizing agents in the liver, thus causing redox imbalance. For instance, exposure to glyphosate has been associated with hepatotoxicity, emerging from the elevation of the levels of reactive oxygen and nitrogen species, ROS and RNS, respectively, and from the impairment of the antioxidant defense mechanisms, events that can potentially drive to oxidative lipid modifications [82,83,84,85]. Similarly, parabens have been found to induce disturbances of liver redox equilibrium due to limitation of antioxidant enzymes activity and depletion of GSH pools, both resulting in lipid peroxidation [86,87]. Hepatic injury mediated by oxidative stress is a common finding in the case of TCS exposure, caused by advanced lipid peroxidation and accumulation of the respective end products [88,89]. Interestingly, BPA administration causes oxidative stress similar to the one noticed in the present study. More specifically, by decreasing CAT activity and increasing TBARS levels [90]. Finally, it has been demonstrated that DEHP administration diminishes GSH reservoirs and impairs the activity of antioxidant enzymes of the GSH pathway [91], even promoting lipid peroxidation in some cases [92].

Contrariwise, the chronic administration of Roundup^®^ activated an adaptive redox response in the liver to alleviate oxidative damage since it appears that the elevation of GSH concentration was induced to counterbalance the reduction in CAT activity. This finding is in agreement with a previous study, wherein the sublethal exposure of Wistar rats for 90 days to Roundup full II decreased CAT activity and increased total glutathione concentration, GSH/GSSG ratio, and GPx activity, acting as a protective mechanism against the potentially detrimental effects of the herbicide [65].

The fact that none of the other examined tissues was affected by the xenobiotics could be largely attributed to the short exposure period. As stated above, the 12-month exposure might not have been sufficient time for the manifestation of effects, since the average life expectancy of New Zealand rabbits is approximately 10 years [41] and, therefore, the exposure time is likely to be short compared to their lifespan.

## 5. Conclusions

**Figure 11:**
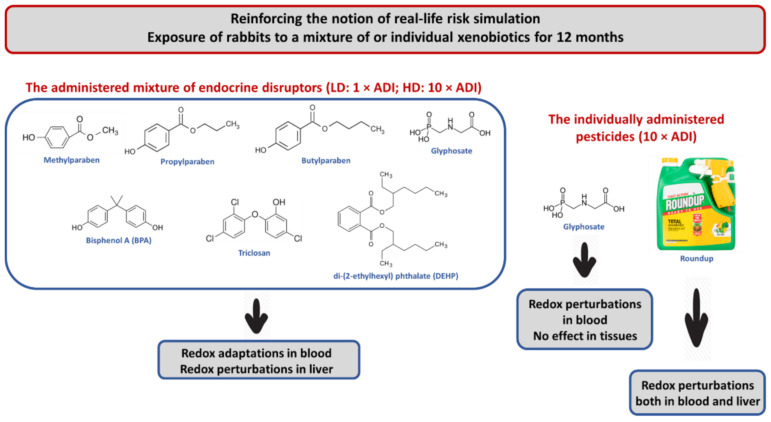
A conceptual illustration of the main findings of the present study. ADI: Acceptable daily intake; LD: Low dose; HD: High dose.

The present investigation reports that at the systemic level (i.e., regarding blood redox biomarkers), the 12-month exposure of the animals to the EDs mixture activated useful redox adaptations. In contrast, glyphosate and Roundup^®^ caused dysregulation of redox homeostasis. Regarding the examined tissues, the 12-month exposure of the animals to the EDs mixture was detrimental for the liver, whose redox equilibrium was adversely affected, resulting in oxidative stress. Roundup^®^ also acted as an oxidizing agent in the liver, whilst glyphosate exerted no adverse effects (Figure 11). In conclusion, the present study reinforces the recently proposed idea of RLRS or the real-life exposure scenario. The mixture of EDs induced harmful outcomes in the liver, an essential tissue for body detoxification when administered in a long-term low-dose regimen.

## Data Availability

The data presented in this study are available on reasonable request from the corresponding author.
